# Supplementary material for: Short- and Long-Term Effects of Inhaled Ultrafine Particles on Blood Pressure: A Systematic Review and Meta-Analysis
Source: J Clin Med. 2024 Nov 12;13(22):6802. doi: 10.3390/jcm13226802 (PMC11594296; doi:10.3390/jcm13226802)
Supplement: Supplementary file 1 [file jcm-13-06802-s001.zip › jcm-3244572-supplementary.pdf]

ELECTRONIC SUPPLEMENTARY MATERIAL

Short-term and long-term effects of inhaled ultrafine particles on blood pressure: A systematic review and meta-analysis

Joanna I. Lachowicz,[1] Pawel Gac[1]

[1]*Department of Population Health, Division of Environmental Health and Occupational Medicine, Wroclaw Medical University, Mikulicza-Radeckiego 7, Wroclaw, PL 50-368, Poland.*

Sommario

Text S1. Search terms used in literature search. 2

Text S2. Standardization of the effect estimates. 3

Table S1. Eligibility criteria of *long-term* exposures on UPFs. 4

Table S2. Eligibility criteria of *short-term* exposures on UPFs. 5

Table S3. Risk of Bias (RoB) assessment prepared according to "Risk of Bias assessment instrument for systematic reviews informing WHO Global Air Quality Guidelines". By: the WHO Global Air Quality Guidelines Working Group on Risk of Bias Assessment. 6

Figure S1. Box plot of mean SBP to mean DBP ratio after short-term and long-term exposure on UFPs. Short-term exposure mean SBP to mean DBP ratio values: mean = 1.571; median = 1.584; IQR = 0.066; Long-term exposure mean SBP to mean DBP ratio values: mean = 1.659; median = 1.722; IQR = 0.233. 8

Table S4. Main characteristics of studies included in the systematic review but not in the meta-analysis. 9

Text S1. Search terms used in literature search.

Search terms used on *PubMed* were: “Nanoparticles" OR "Ultrafine particles" AND “Cardiovascular” AND “Exposure”.

Search terms used on *Web of Science* were: “Nanoparticles" OR "Ultrafine particles" AND “Cardiovascular” AND “Exposure”.

Search terms used on *Embase* were: “Nanoparticles" OR "Ultrafine particles" AND “Cardiovascular” AND “Exposure”.

Search terms used on *Scopus* were: “Nanoparticles" OR "Ultrafine particles" AND “Cardiovascular” AND “Exposure”.

Text S2. Standardization of the effect estimates.

When BP indices were not transformed:

$$\text{Percent change} = \beta \,/\, \text{mean} \times 100\%$$

$$95\%\ \text{CI} = (\beta \pm 1.96\times\text{SE}) \,/\, \text{mean} \times 100\%$$

β is the estimated regression coefficient increase in UFPs (expressed in PNC), *SE* is the standard error of *β*, *mean* is the arithmetic mean of the examined BP index (SBP or DBP).

We chose transformation to percent changes in the geometric mean because we were not able to calculate percent changes in the arithmetic mean in all studies due to missing geometric or/and arithmetic mean of the BP indices. The procedure above enabled us to include all eligible articles in meta-analyses.

Table S1. Eligibility criteria of *long-term* exposures on UPFs.

PECOS	Inclusion	Exclusion
**P**opulation	General human population (including subgroups: children, women, man) of all ages, living in developed and developing areas, both urban and rural. No geographical restrictions	No exclusion criteria applied
	Exposure to UFPs via inhalation through ambient air (this covers exposures in both outdoor and indoor environments)	
**E**xposure	Long-term exposure (in the order of months to years) to ambient air UFPs a concentration unit (particles/m^3)	No exclusion criteria applied
	Additionally, Long-term exposure to other air pollutants	
**C**omparator	Exposure to lowest levels of the air UFPs in the same or a control population	No exclusion criteria applied
**O**utcome	Health outcomes selected in relation to long-term exposure include: systolic blood pressure, diastolic blood pressure	No exclusion criteria applied
**S**tudy design	Human epidemiological studies such as:<br>- panel studies<br>- cohort studies<br>- cross-sectional studies<br>- cross-over studies<br>- Published (or accepted for publication, i.e. in press) studies in peer-reviewed indexed journals in any language (abstract in English language)	Qualitative studies; Studies without individual- level data, that is, fully group-level (ecological) covariates; Reviews and methodological papers; Non-human studies (in vivo, in vitro, other);

Table S2. Eligibility criteria of *short-term* exposures on UPFs.

PECOS	Inclusion	Exclusion
**P**opulation	General human population (including subgroups: children, women, man) of all ages, living in developed and developing areas, both urban and rural. No geographical restrictions	No exclusion criteria applied
	Exposure to UFPs via inhalation through ambient air (this covers exposures in both outdoor and indoor environments)	
**E**xposure	Short-term exposure (in the order of hours to 7 days) to ambient air UFPs a concentration unit (particles/m^3)	No exclusion criteria applied
	Additionally, short-term exposure to other air pollutants	
**C**omparator	Exposure to lowest levels of the air UFPs in the same or a control population	No exclusion criteria applied
**O**utcome	Health outcomes selected in relation to short-term exposure include: systolic blood pressure, diastolic blood pressure	No exclusion criteria applied
**S**tudy design	Human epidemiological studies such as:<br>- panel studies<br>- cohort studies<br>- cross-sectional studies<br>- cross-over studies<br>- Published (or accepted for publication, i.e. in press) studies in peer-reviewed indexed journals in any language (abstract in English language)	Qualitative studies; Studies without individual-level data, that is, fully group-level (ecological) covariates; Reviews and methodological papers; Non-human studies (in vivo, in vitro, other);

Table S3. Risk of Bias (RoB) assessment prepared according to "Risk of Bias assessment instrument for systematic reviews informing WHO Global Air Quality Guidelines". By: the WHO Global Air Quality Guidelines Working Group on Risk of Bias Assessment.

	Confounding				Selection bias		Exposure assessment					Outcome measurement				Missing data		
Study	Were all confounders considered adjusted for in the analysis?	Validity of measuring of confounding factors	Control in analysis (Did the authors use an appropriate analysis method or study design [illegible]	Overall	Selection of participants into the study (includes non-response)	Overall	Methods used for exposure assessment	Exposure measurement methods comparable across the range of exposure	Change in exposure status (for long-term studies only)	Exposure contrast	Overall	Blinding of outcome measurement	Validity of outcome measurements	Outcome measurement	Overall	Missing data of outcome measures	Missing data of exposures	Overall
Liu et al.	low	low	low	low	some concerns	some concerns	low	some concerns	NA	low	low	some concerns	low	low	low	low	low	low
Weichenthal et al.	low	low	low	low	low	low	low	low	NA	low	low	low	low	low	low	some concerns	low	low
Devlin et al.	low	low	low	low	low	low	low	low	NA	low	low	low	low	low	low	low	low	low
Meier et al.	low	low	low	low	some concerns	some concerns	low	low	NA	low	low	low	low	low	low	low	low	low
Chung et al.	some concerns	some concerns	some concerns	some concerns	low	low	some concerns	some concerns	NA	some concerns	some concerns	low	low	low	low	low	low	low
Kubesh et al.	low	low	low	low	low	low	low	low	NA	low	low	low	low	low	low	low	low	low
Pieters et al.	low	low	low	low	some concerns	some concerns	low	low	NA	low	low	some concerns	low	low	low	low	low	low
Padró-Martínez et al.	some concerns	some concerns	some concerns	some concerns	high	high	moderate	low	NA	some concerns	some concerns	low	low	low	low	low	low	low
Soppa et al.	low	low	low	low	low	low	low	some concerns	NA	low	low	some concerns	low	low	low	some concerns	some concerns	some concerns

Chen et al.	low	low	low	low	low	low	some concerns	some concerns	NA	some concerns	some concerns	low	low	low	low	low	low	low
Corlin et al.	high	some concerns	some concerns	some concerns	low	low	some concerns	high	high	some concerns	some concerns	low	some concerns	low	low	some concerns	some concerns	some concerns
Liu et al.	low	low	low	low	low	low	low	low	low	low	low	low	low	low	low	low	low	low
Schubauer-Berigan et al.	low	low	low	low	low	low	low	low	NA	low	low	low	low	low	low	low	low	low
Gabdrashova et al.	low	low	low	low	low	low	some concerns	low	NA	low	low	some concerns	low	low	low	low	low	low
Lin et al.	high	high	high	high	low	low	some concerns	some concerns	NA	some concerns	some concerns	low	low	low	low	low	some concerns	some concerns
Lyu et al.	some concerns	low	low	low	low	low	low	low	NA	low	low	some concerns	low	low	low	low	low	low
Guo et al.	some concerns	low	low	low	low	low	low	low	NA	low	low	low	low	low	low	low	low	low
Roswall et al.	low	low	low	low	low	low	some concerns	some concerns	some concerns	some concerns	some concerns	low	low	some concerns	low	low	low	low
Gilbey et al.	low	low	low	low	some concerns	some concerns	low	low	NA	low	low	low	low	low	low	low	low	low

Figure S1. Box plot of mean SBP to mean DBP ratio after short-term and long-term exposure on UFPs. Short-term exposure mean SBP to mean DBP ratio values: mean = 1.571; median = 1.584; IQR = 0.066; Long-term exposure mean SBP to mean DBP ratio values: mean = 1.659; median = 1.722; IQR = 0.233.

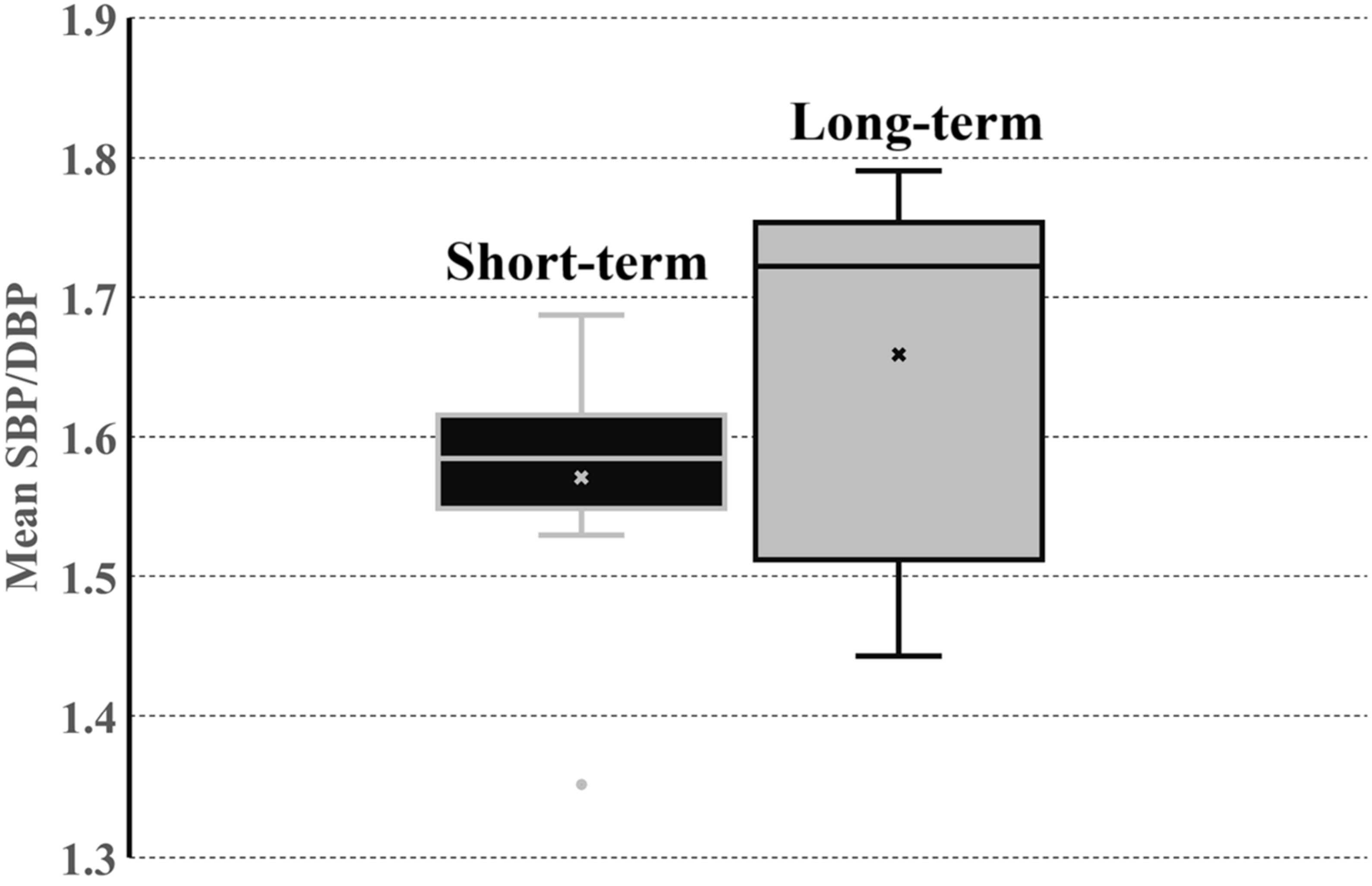

Table S4. Main characteristics of studies included in the systematic review but not in the meta-analysis.

No	Study	Area and period	Population/sample size	Experiment type	Exposure assesment	Mean ± SD (range) ($\times 10^3$ particles/cm^3)	Statistics	Estimate (95% CI)	Reason for exclusion
1	Devlin et al. [13]	campus of the University of North Carolina, US	34 middle-aged individuals with metabolic syndrome (13 male and 21 female)	laboratory exposure chamber	monitored in real time	100	linear mixed effects models	**Short-term outcome:**<br>%change in SBP: -0.20(0.77) after 1 h; -0.85(0.70) afre 20 h<br>%change in DBP: -0.81(0.87) after 1 h; -0.63 (0.88) after 20 h	All data obtained on individuals with metabolic syndrom
2	Pieters et al. [10]	Belgium, Antwerp; spring (17 May–20 June) and fall (10 November–13 December) 2011	130 healthy children (6–12 years of age)	real environment	stationery particle counter	5.538 (25th percentile); 7.204 (75th percentile)	mixed models with random subject effects	**Long-term outcome:**<br>SBP increase of 0.79 mmHg (95% CI: 0.07, 1.51; p = 0.03)	Studies on children; the obtained results significantly different than data obtained on adults due to physiological reason
3	Chen et al. [17]	China, Beijing; winter 2014	20 healthy non-smoking male subjects (age ranging 18–26 years)	real environment	fast mobility particle sizer	16.2(7.3)	Linear mixed-effects models	**Long-term outcome:**<br>Increased SBP was associated with 5- and 7-d MA exposure to accumulation mode particles (β = 1–2%).	Insufficient analytical data for meta-analysis
4	Schubauer-Berigan et al. [25]	US; 12/2012-9/2014	108 workers of carbon nanotubes industry	Occupational environment	personal monitoring	6.22(41.2)[ug/m^3]	univariable linear regression modeling	**Short-term outcome:**<br>SBP (CNT/F duration emp; β estimate (p)) = 0.512 (0.1353); DBP (CNT/F duration emp; β estimate (p)) = 0.0788 (0.7486)	Insufficient analytical data for meta-analysis
5	Gabdrashova et al. [14]	Kazakhstan, Nur-Sultan	Phase 1: 17 healthy non-smoking adults (7 men and 10 women, aged 18 - 46 years); Phase 2:33 non-smoking healthy adults (11 men and 21 women, aged 18 - 51 years)	Laboratory	condensation Particle Counter	Phase 1: 5; Phase 2: 20	The Friedman test and Wilcoxon Test as our post-hoc test	**Short-term outcome:**<br>Phase 1: average SBP increased from 105.0 ± 10.0 mm Hg before cooking to 109.0 ± 8.6 mm Hg and 109.0 ± 9.0 mm Hg after 90- and 120-min post-exposure, respectively Phase 2: SBP fluctuated with a statistically significant increase from 98.8 ± 8.5 (before cooking) to 102.0 ± 10.0 mm Hg (p = 0.02) 60 min after the end of cooking, followed by a statistically significant decrease to 98.8±10.5mmHg (p=0.006) at 90min post-exposure, and a non-statistically significant increase to 102.2 ± 9.5 mm Hg at 120 min post-exposure	Insufficient analytical data for meta-analysis
6	Lin et al. [28]	Northeast China; 2006–2009	24 845 adults (aged 18–74 years)	real environment	chemical transport model	5.9 ± 0.8 μg m^{-3} with the range of 4.5–6.8 μg m^{-3}	one-way analysis-of-variance test for continuous variables and chi-square tests for categorical variables	**Long-term outcome:**<br>increase in SBP of 1.52 mm Hg [95% confidence interval (CI): 0.48–2.55], and in DBP of 0.55 mm Hg (95% CI: 0.01–1.08) in DBP.	Insufficient analytical data for meta-analysis

7	Lyu et al. [21]	China, Beijing	53 printing room workers and 54 controls, age >16 years	real environment	stationery particle counter	workplace: 9.968 ± 4.665, control: 4.667 ± 1.840	unpaired Student's t-tests; Multiple regression analysis	**Short-term outcome:** Unpaired test t-student (exposed vs. unexposed workers): SBP: -3.36 (p = 0.001); DBP: -3.01 (p = 0.003)	Insufficient analytical data for meta-analysis
